# Sustained Local Diversity of *Vibrio cholerae* O1 Biotypes in a Previously Cholera-Free Country

**DOI:** 10.1128/mBio.00570-16

**Published:** 2016-05-03

**Authors:** Yan Boucher

**Affiliations:** Department of Biological Sciences, University of Alberta, Edmonton, Alberta, Canada

## Abstract

Although the current cholera pandemic can trace its origin to a specific time and place, many variants of *Vibrio cholerae* have caused this disease over the last 50 years. The relative clinical importance and geographical distribution of these variants have changed with time, but most remain in circulation. Some countries, such as Mexico and Haiti, had escaped the current pandemic, until large epidemics struck them in 1991 and 2010, respectively. Cholera has been endemic in these countries ever since. A recent retrospective study in *mBio* presents the results of more than 3 decades of *V. cholerae* monitoring from environmental and clinical sources in Mexico (S. Y. Choi et al., mBio 7:e02160-15, 2016, http://dx.doi.org/10.1128/mBio.02160-15). It reveals that multiple *V. cholerae* variants, including classical strains from the previous pandemic, as well as completely novel biotypes, have been circulating in Mexico. This discovery has important implications for the epidemiology and evolution of *V. cholerae*.

## COMMENTARY

Cholera is an ancient and often fatal diarrheal disease, which still affects 3 to 5 million people a year, causing around 120,000 deaths (1). Its causative agent, *Vibrio cholerae*, is an aquatic bacterium with various environmental reservoirs. Its ability to survive in both freshwater and saltwater environments and its association with various zooplanktons and phytoplanktons make it easy for this species to be dispersed with ships or currents. As a significant proportion of carriers are asymptomatic, *V*. *cholerae* can also be spread by unsuspecting travelers. There have been six cholera pandemics between 1817 and 1923, all caused by *V*. *cholerae* of the O1 serogroup and classical biotype, which likely originated from southern Asia (1). We are currently experiencing a seventh pandemic of cholera, which started in 1961 in Indonesia and is also caused by *V*. *cholerae* O1, but of a different biotype, named El Tor from its original discovery in El Tor, Egypt (2).

New variants of this prototype *V. cholerae* O1 El Tor have emerged regularly during the ongoing pandemic. These variants include strains that, within an El Tor genetic backbone, harbor classical biotype features and/or other divergent genetic features and hence are named atypical El Tor. They are often distinguished from prototype strains by various phenotypic and genotypic traits, including mutations in genes encoding the two major virulence factors of *V. cholerae*, the cholera toxin and the toxin-coregulated pilus (3). After the initial wave of the current pandemic spread prototype El Tor strains across the world, two additional waves spread the atypical El Tor strains derived from these prototype strains, each wave mostly displacing bacteria from the preceding one (4). Interestingly, the novel strain responsible for each wave always originated from the Bay of Bengal (5), where cholera has been endemic for centuries (6). The classical biotype responsible for previous pandemics has been increasingly rare, and it has not been isolated from Asia or Africa after 1992 (7). A recent series of retrospective studies have reported the discovery of classical, prototype El Tor, atypical El Tor, and nontoxigenic O1 strains with some unusual genetic features in Mexico from 1983 to 2008 (7–10). The latest of these studies, recently published in *mBio*, reports the comparative genomic and phylogenetic analysis of these various biotypes (8).

Such a high diversity of biotypes from a short time period in a single country is highly unexpected. When areas previously free of cholera experience epidemics, such as that which started in Peru in 1991 and spread across Latin America, they are usually attributed to a single imported strain. The finding of multiple, simultaneously occurring biotypes calls into question not only our understanding of *Vibrio cholerae* O1 strain importation into regions where cholera had previously not been endemic but also their long-term survival and diversification in these environments. It also has concerning implications for the recent importation of cholera in Haiti and the future of this disease in Latin American ecosystems.

## HOW TO INTERPRET THE STRIKING BIOTYPE DIVERSITY OF MEXICAN *V. CHOLERAE* ISOLATES?

The article (8) describes a rare instance where a large variety of O1 strains are found to cohabitate outside of southern Asia. This diversity is remarkable because cholera was absent from Mexico for at least a hundred years until the 1991 Latin American epidemic. This epidemic was mainly attributed to prototype El Tor and was proposed to have originated in Africa, because of similarity of Latin American isolates to African strains (5). The study presented in *mBio* (8) found a range of both prototype and atypical El Tor strains as early as 1991, some of which present no specific similarity to African strains. This casts serious doubt on the theory of a single African source origin for this epidemic. Interestingly, both prototype and atypical El Tor isolates were found from 1991 to 2000, while only prototype El Tor isolates were identified through 2008 (the end of the study period). This clearly does not match the pattern observed elsewhere around the world, in which prototype El Tor has been increasingly rare after 1991, and suggests a unique local population dynamic for *V. cholerae* O1 in Mexico. Also of note is the presence of classical strains in Mexico from 1983 to 1997, which are closely related to those previously found in Asia and Africa. Classical strains were thought to be extinct even from their last known habitat in the Bay of Bengal region since 1992, but their continued presence in Mexico for more than a decade underscores their resilience and indicates that this biotype might not be extinct today. These classical strains could be responsible for the presence of atypical El Tor strains with classical features in Mexico, if horizontal gene transfer to imported prototype El Tor occurred.

It is likely that the strains found in Mexico are a mixture of indigenous and imported strains. Those closely related to strains found in southern Asia or other parts of the world (El Tor, atypical El Tor, and classical) were most likely imported to Mexico, possibly through human hosts or the ballast water of ships (11). The timing of importation of these strains is undefined at this point and is complicated by the fact that some strains certainly evolved locally after introduction in Mexico. Given the diversity of strains found, there were likely multiple imports from different sources at different times, a statement supported by the characteristics of *V. cholerae* strains from recent cholera cases in Mexico (12).

## WHAT CLUES DOES THIS STUDY PROVIDE TO THE O1 PANDEMIC LINEAGE ANCESTRY?

**FIG 1:**
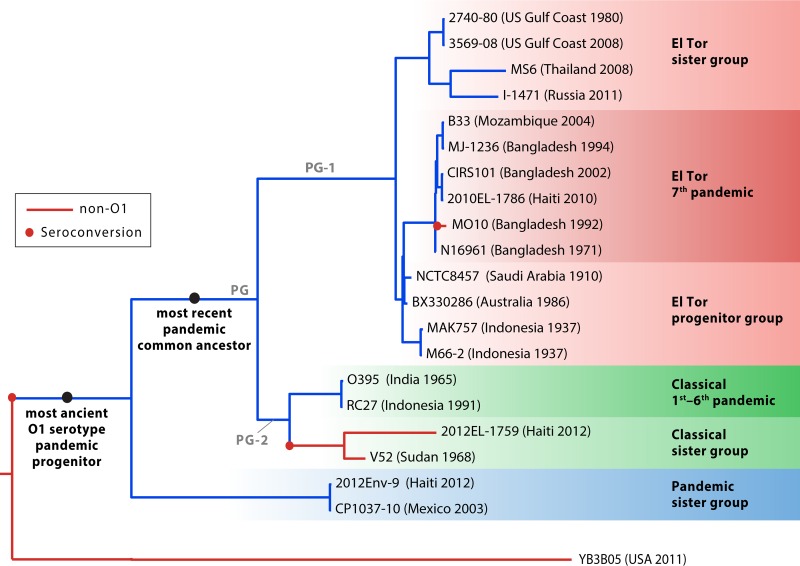
Phylogenetic relationships of pandemic *Vibrio cholerae* strains and their relatives. PG is an abbreviation for phylocore genome/pandemic group.

Among the *V. cholerae* O1 bacteria discovered in Mexico was a strain which is a clear sister group basal to all classical and El Tor strains (Fig. 1). This finding is key to retracing the origin(s) of pandemic strains. The novel O1 strain type shares numerous phenotypic and genotypic characteristics with El Tor and classical isolates likely derived from the recent common ancestor they share. This “pandemic sister” taxon lacks a CTX prophage, which encodes genes for the cholera toxin. However, it belongs to the O1 serogroup and possesses a *Vibrio* pathogenicity island 1 (VPI-1) encoding the toxin-coregulated pilus, which allows attachment in the intestine and is the receptor for the CTX prophage. This suggests that the progenitor of classical and El Tor strains was first seroconverted to the O1 serogroup and acquired VPI-1 before incorporating the CTX prophage which allows the production of cholera toxin. This pandemic sister group is possibly indigenous to Mexico, and its representatives could be more widespread geographically.

## IS THE *V. CHOLERAE* O1 DIVERSITY FOUND IN MEXICO THE EXCEPTION OR THE RULE?

The sampling of *V. cholerae* O1 performed in Mexico (8) is exceptional for an area where cholera has not been endemic for a long period of time. Isolates were collected from both clinical and environmental sources for more than 2 decades and carefully typed for all known characteristics of pandemic strains. Had such an extensive effort been conducted around other major epidemics in countries not known for the continuous presence of cholera, would a similar diversity have been found? The recent Haiti epidemic, which started in 2010, has triggered substantial sampling and monitoring efforts both in clinical and environmental settings. The cause of this epidemic is now well-known as an atypical El Tor strain circulating in southern Asia imported to Haiti by United Nations (UN) troops from Nepal (13). No prototype El Tor or classical strains have been found in Haiti, suggesting that such strains might not be circulating freely around South America and that their sustained presence in Mexico could be related to local factors. Further investigation of other South American locations is needed to establish the distribution patterns of these diverse strains and whether they have taken hold more broadly.

Interestingly, other pandemic-related strains have now been found in Haiti as a result of the extensive environmental sampling that has taken place since the start of the epidemic. A strain isolated from water in Haiti in 2012 is very closely related to the Mexican *V. cholerae* O1 strain basal to the classical and El Tor pandemic biotypes (Fig. 1) (14). This suggests that strains from this pandemic sister group could be circulating more broadly around South America. A non-O1 strain related to classical biotype strains was also found in environmental samples in Haiti (15). This strain is reminiscent of *V. cholerae* O37 strain V52 isolated from Sudan 1968 and clearly clusters with classical biotype strains despite significant evolutionary divergence and harboring a different serogroup (Fig. 1). Because of the genetic backbone they share with classical and El Tor biotypes, these unusual pandemic-related strains could be progenitors for novel variants able to initiate cholera outbreaks. Their discovery in Haiti and Mexico suggests that they could be widespread and that large environmental sampling efforts are needed to detect their presence.
